# Supplementary material for: Lymphovascular Space Invasion in Early-Stage Endometrial Cancer (LySEC): Patterns of Recurrence and Predictors. A Multicentre Retrospective Cohort Study of the Spain Gynecologic Oncology Group
Source: Cancers (Basel). 2023 May 4;15(9):2612. doi: 10.3390/cancers15092612 (PMC10177148; doi:10.3390/cancers15092612)
Supplement: Supplementary file 1 [file cancers-15-02612-s001.zip › cancers-2318152-supplementary.pdf]

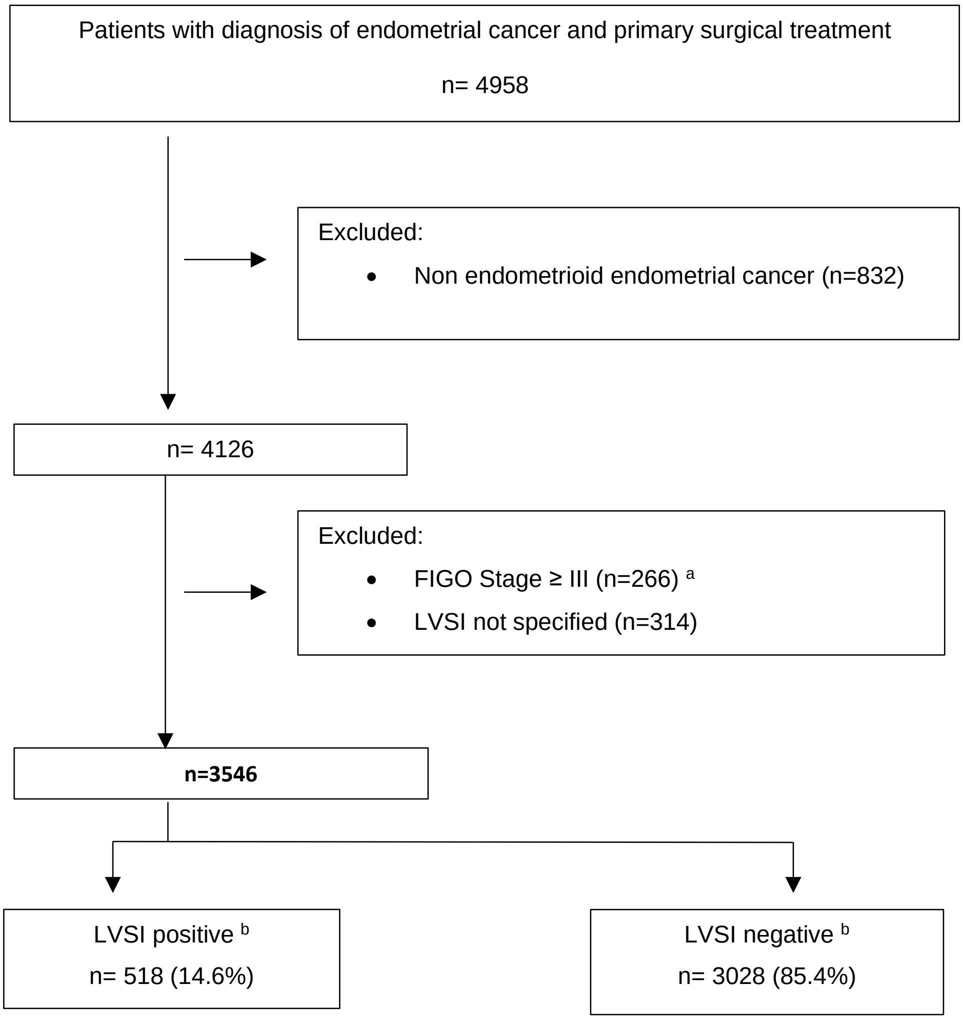

Figure S1. Flow chart of the study population. [a] FIGO 2009 (18) [b] LVSI positive: diffuse or multifocal presence of tumor cells inside a space surrounded by endothelial cells detected on hematoxylin-eosin-stained sections (substantial LVSI). LVSI negative: No or focal LVSI (4). *LVSI,* Lymohovascular Space Involvement; *FIGO*, Federation of Gynaecology and Obstetrics.
